# Simultaneous Estimation of Metformin Hydrochloride and Pioglitazone Hydrochloride by RPHPLC Method from Combined Tablet Dosage Form

**DOI:** 10.4103/0250-474X.43010

**Published:** 2008

**Authors:** P. K. Sahoo, R. Sharma, S. C. Chaturvedi

**Affiliations:** School of Pharmacy, Devi Ahilya Vishwa Vidyalaya, Takshashila Campus, Khandwa Road (Ring Road), Indore-452 017, India

**Keywords:** Pioglitazone hydrochloride, metformin hydrochloride, reverse-phase, simultaneous estimation

## Abstract

A high performance reverse phase liquid chromatographic procedure is developed for simultaneous estimation of metformin hydrochloride and pioglitazone hydrochloride in combined tablet dosage form. The mobile phase used was a combination of acetonitrile:water:acetic acid (60:40:0.3) and the pH was adjusted to 5.5 by adding triethylamine. The detection of the combined dosage form was carried out at 230 nm and a flow rate employed was 1 ml/min. Linearity was obtained in the concentration range of 0.015 to 0.120 μg/ml of pioglitazone hydrochloride and 0.5 to 4.0 μg/ml of metformin hydrochloride with a correlation coefficient of 0.9992 and 0.9975. The results of the analysis were validated statistically and recovery studies confirmed the accuracy and precision of the proposed method.

Metformin hydrochloride (MET) is an oral antidiabetic drug and is chemically N,N-dimethyl imidodicarbonimidic diamide. Few UV Spectrophotometric methods^1^–^2^, HPLC 3–7 and ion-pair HPLC^8^ method have been reported for the estimation of MET. Pioglitazone hydrochloride (PIO) is a member of type-2 oral antidiabetic agents called thiazolidinediones or insulin sensitizers, which makes body more sensitive to insulin. Chemically PIO is (±)-5-[4-[2-(5-ethyl-2-pyridinyl) ethoxy]phenyl]methyl]-2,4-thiazolidinedione^1^. PIO is not yet official in any of the pharmacopoeia but MET is official in IP^9^, BP^10^ and USPNF^11^. Literature indicated an RPHPLC method and a MEKC method, for the determination of PIO from plasma as well as pharmaceutical preparations^1^. The review of the literature revealed that no RPHPLC method is not reported for the simultaneous estimation of the PIO and MET in combined pharmaceutical dosage form. Therefore, it was thought worthwhile to develop a simple, precise, accurate reverse phase high performance liquid chromatographic method for the simultaneous estimation of PIO and MET in combined tablet dosage form.

Pharmaceutical grade PIO (Material Code: 2000515 and Batch No.: 1597684) and MET (Material Code: 3002173 and Batch No.: 1689373) were kindly supplied as a gift sample by Ranbaxy Laboratories Limited, Dewas-455 001, India. The tablet dosage form (Pioglar-M, Batch No. 1670867, Mfg. Dt. 07/2006 and Exp. Dt. 06/2008) was procured from a local pharmacy (Label claim: 15 mg of PIO and 500 mg of MET) marketed by Ranbaxy Laboratories Limited, Ponda, Goa-403 404, India. All chemicals used were of HPLC grade and were purchased from Spectrochem, Mumbai, India. LC system used consist of pump (Model Shimadzu; LC-10 AT VP) with universal loop injector (Rheodyne 7725) of injection capacity 20 μl. Detector consists of photodiode array detector SPD-10 AVP, Shimadzu; the reversed phase column used was Luna C_18_ (5mm, 25cm×4.6 mm i.d.) phenomenex, USA, at ambient temperature.

Among the several mobile phases used for the simultaneous estimation of PIO and MET, acetonitrile:water:acetic acid (60:40:0.3) ratio was found to be most suitable and the pH was adjusted to 5.5 by adding triethylamine and was filtered through 0.2 micron membrane filter.

Standard stock solutions of PIO and MET were prepared by dissolving 5 mg of each in methanol and the volume were made up to 10 ml with mobile phase. From the above stock solutions dilutions were made in the concentration range of 0.015 to 0.120 μg/ml of PIO and 0.5 to 4.0 μg/ml of MET. All solutions were stored at room temperature. Each standard solution (20μl) was injected into the column after filtration using 0.2 micron membrane filter. All measurements were repeated five times and the calibration curves were constructed by plotting the peak area versus the corresponding drug concentration. The slope and correlation coefficients were determined, which were found to be 0.9992 for PIO and 0.9975 for MET.

To determine the content of PIO and MET in tablet dosage form; twenty tablets were weighed; their average weight was determined and were finely powdered. Then 54.3 mg of triturate tablet dosage form was taken which is equivalent to 0.75 mg of PIO and was dissolved in 2 ml of methanol by stirring for 2 min. and the volume was made up to 10 ml using mobile phase. Then 1 ml from that solution was taken and diluted with mobile phase to make up to 10 ml. Again 2 ml from the later solution was taken and diluted with mobile phase to make up to 10 ml. The final solution was filtered using 0.2-micron membrane filter and using an injection filter. Then with the help of 1000 μl micropipette 10, 20, 30, 40, 50, 60, 70, and 80 μl of the filtered solution was taken in small test tubes and diluted up to 1000 μl of with the mobile phase, which contain 0.5:0.015, 1.0:0.030, 1.5:0.045, 2.0:0.060, 2.5:0.075, 3.0:0.090, 3.5:0.105, and 4.0:0.120 μg/ml of both the drugs. 20 μl of the above dilutions were injected one by one to the HPLC with the help of Hamilton Syringe. The results are presented in Table 1.

**TABLE 1:** RESULTS OF ANALYSIS OF TABLET FORMULATION

DRUG	Conc. taken (μg)	Conc. found (μg)*	S.D.	% RSD
MET	1.0	1.0021	0.0015	0.2
	2.0	2.0010	0.0027	0.1
	3.0	3.0000	0.0028	0.1
	4.0	3.9989	0.0034	0.1
PIO	0.030	0.0302	0.0054	0.1
	0.060	0.0601	0.0033	0.2
	0.090	0.0900	0.0029	0.2
	0.120	0.1198	0.0015	0.1

Conc.: Concentration, SD: standard deviation

% RSD: percent relative standard deviation

* Results are mean five replications.

The HPLC method was found to be simple, accurate, economic and rapid for routine simultaneous estimation of PIO and MET, in combined tablet dosage form. The regression: 0.9992 and 0.9975, intercept:–222 and –3164 and slope: 671933 and 987374 were found to be for PIO and MET, respectively. Recovery was in the range of 99-101%; the value of standard deviation and percentage relative standard deviation were found to be less than 2%; shows the high precision of the developed method (Tables 1 and 2).

**TABLE 2:** RESULTS OF RECOVERY STUDIES

Qty. Taken PIO (μg)	Qty. taken MET (μg)	Qty. added PIO (μg)	Qty. Added MET (μg)	% recovery PIO	% recovery MET
0.030	1.0	0.015	0.5	99.55	100.05
0.030	1.0	0.030	1.0	100.50	100.12
0.030	1.0	0.045	1.5	100.66	100.00
0.060	2.0	0.015	0.5	100.26	100.02
0.060	2.0	0.030	1.0	99.77	99.95
0.060	2.0	0.045	1.5	100.09	100.02

Qty.: Quantity. Results are mean of five replicates; percentage recovery is more than 99%. Hence the method is accurate and precise

In proposed method, HPLC conditions were optimized to obtain, better separation of eluted compounds. Amongst the various mobile phases used, acetonitrile:water:acetic acid in 60:40:0.3 ratio and the pH adjusted to 5.5 by the addition of triethylamine was found robust with 1 ml/min. flow rate. Mobile phase and flow rate selection was based on peak parameters such as height, tailing, theoretical plates, capacity factor, run time, resolutions etc. A typical chromatogram of PIO and MET is shown in (fig. 1). The optimum wavelength for detection was 230 nm at which detector response was best obtained. The average retention time for PIO and MET was found to be 5.35±0.05 min and 2.150±0.05 min, respectively. According to USP XXIV (621)^12^, system suitability tests are an integral part of chromatographic method. They are used to verify reproducibility of the chromatographic system. To ascertain its effectiveness, system suitability tests were carried out and its results are shown in Table 3.

**Fig. 1:**
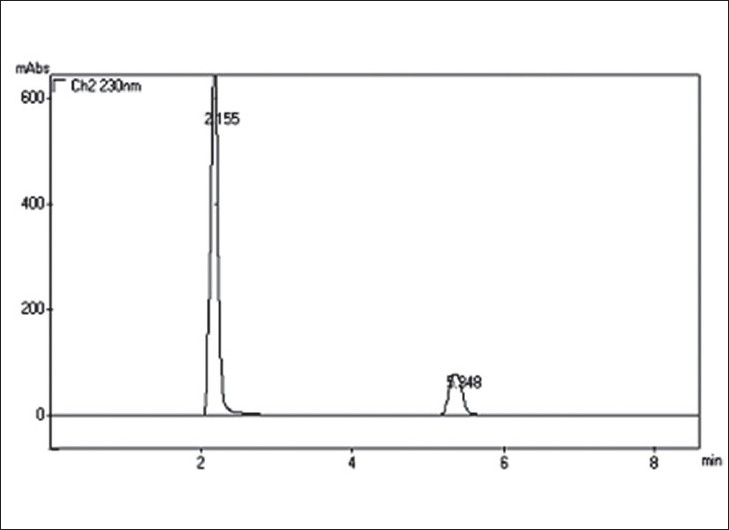
Typical Chromatogram of MET and PIO. Chromatogram showing retention time, 2.155 and 5.348 for MET and PIO in tablet dosage form, respectively.

**TABLE 3:** RESULTS OF VALIDATION STUDIES

SST and other parameters	MET	PIO
*Theoretical Plates (N)	2397	5272
*Resolution (R_s_)	--	13.57
Linearity Range (μg/ml)	0.5-4.0	0.015-0.12
Percentage Recovery (%)	99.95	99.82
LOD (μg/ml)	0.001	0.007
LOQ (μg/ml)	0.002	0.002
*Tailing Factor	1.28	1.05
*Capacity Factor	--	1.49
*Retention Time (Minutes)	2.155	5.348
Standard Deviation	1.9896	0.1507
% RSD	0.3978	1.007
Slope (m) in Tablet	987374	671933
Intercept (b) in Tablet	-3164	-222
Co-relation coefficient	0.9975	0.9992

* Calculated at 5% peak height.

SST: system suitability test, LOD: limit of detection, LOQ: limit of quantitation.
